# BIM and Data-Driven Predictive Analysis of Optimum Thermal Comfort for Indoor Environment

**DOI:** 10.3390/s21134401

**Published:** 2021-06-27

**Authors:** Vincent J.L. Gan, Han Luo, Yi Tan, Min Deng, H.L. Kwok

**Affiliations:** 1 School of Design and Environment, National University of Singapore, Singapore 117566, Singapore; vincent.gan@nus.edu.sg; 2 Department of Civil and Environmental Engineering, Hong Kong University of Science and Technology, Hong Kong, China; han.luo@connect.ust.hk (H.L.); hlkwokab@connect.ust.hk (H.L.K.); 3 Sino-Australia Joint Research Centre in BIM and Smart Construction, Shenzhen University, Shenzhen 518060, China; 4 Department of Civil and Environmental Engineering, University of Michigan, Ann Arbor, MI 48109, USA; mindeng@umich.edu

**Keywords:** building information modelling, energy conservation, machine learning, natural ventilation, optimization, thermal comfort

## Abstract

Mechanical ventilation comprises a significant proportion of the total energy consumed in buildings. Sufficient natural ventilation in buildings is critical in reducing the energy consumption of mechanical ventilation while maintaining a comfortable indoor environment for occupants. In this paper, a new computerized framework based on building information modelling (BIM) and machine learning data-driven models is presented to analyze the optimum thermal comfort for indoor environments with the effect of natural ventilation. BIM provides geometrical and semantic information of the built environment, which are leveraged for setting the computational domain and boundary conditions of computational fluid dynamics (CFD) simulation. CFD modelling is conducted to obtain the flow field and temperature distribution, the results of which determine the thermal comfort index in a ventilated environment. BIM–CFD provides spatial data, boundary conditions, indoor environmental parameters, and the thermal comfort index for machine learning to construct robust data-driven models to empower the predictive analysis. In the neural network, the adjacency matrix in the field of graph theory is used to represent the spatial features (such as zone adjacency and connectivity) and incorporate the potential impact of interzonal airflow in thermal comfort analysis. The results of a case study indicate that utilizing natural ventilation can save cooling power consumption, but it may not be sufficient to fulfil all the thermal comfort criteria. The performance of natural ventilation at different seasons should be considered to identify the period when both air conditioning energy use and indoor thermal comfort are achieved. With the proposed new framework, thermal comfort prediction can be examined more efficiently to study different design options, operating scenarios, and changeover strategies between various ventilation modes, such as better spatial HVAC system designs, specific room-based real-time HVAC control, and other potential applications to maximize indoor thermal comfort.

## 1. Introduction

Buildings account for 40% of the carbon emissions and a third of the energy consumption in the world [1]. In subtropical cities, buildings account for as much as 61% of carbon emissions and 90% of electricity consumption, both much higher than the worldwide average [2]. The use of a mechanical air conditioning system is one of root cause for the high electricity use [3]. Mechanical ventilation mainly aims at providing occupants with good indoor thermal comfort. In addition to mechanical ventilation, natural ventilation, which is more energy-saving and environmentally friendly, is also able to contribute to good thermal comfort. Previous studies have shown that natural ventilation can help maintain occupant thermal comfort while decreasing the need for mechanical ventilation and therefore the electricity consumption for a large portion of the year [4,5].

Many studies of the use of natural ventilation to improve indoor thermal comfort have been conducted. For example, Han et al. [6] compared the thermal comfort between urban and rural residential areas under ventilation environments in central southern China. Natural ventilation has also been applied by different countries, such as the UK, Singapore, and India [7,8,9], for thermal comfort in residential buildings. According to the existing ventilation studies, computational fluid dynamics (CFD) is one of the efficient techniques for airflow modelling and simulation in a ventilated environment. For example, Song and Meng [10] integrated CFD simulation with a field measurement study of the boundary conditions to enhance the natural ventilation in a classroom. D′Agostino et al. [11] used CFD to model the effect of ventilation strategies on the microclimate of certain spaces. Several studies have been conducted to assess the effect of window openings on indoor air ventilation, such as single-sided and cross ventilation [12,13]. Furthermore, Micallef et al. [14] evaluated how the courtyard height affects the cross-ventilation of a room abating a courtyard building using CFD. D’Agostino and Congedo [15] developed a CFD model to investigate the adequacy of natural ventilation in a historical building from the south of Italy. Gou et al. [16] used CFD to optimize a building’s natural ventilation from three aspects, namely site planning, building shape, and building envelope.

Predicting thermal comfort of building occupants can not only ensure comfortable living environments, but also improve building energy efficiency according to the thermal state index of occupants. However, CFD simulation is computationally very demanding, and the conventional method of evaluating thermal comfort (using mathematical models) is an iterative and time-consuming process; thus, developing a more efficient approach to underpin the evaluation of thermal comfort becomes important. Chaudhuri et al. [17] presented a TSI prediction method (called enhanced predictive thermal state method), which can predict by sensing physiological parameters (hand skin temperature and pulse frequency) and ambient air temperature. In addition, based on an optimized support vector machine algorithm, six important TSI prediction features were proposed. Mocho et al. [18] improved the performance of box model prediction (predicting indoor air formaldehyde concentration) by testing a wide range of material surface concentrations and ventilation conditions in a rectangular parallelepiped geometry room to implement CFD simulations. In order to capture the complex and highly subjective relationship between room conditions and thermal perception, Guenther and Sawodny [19] proposed a comfort model based on a polynomial basis function extended from a Gaussian process regression model to evaluate the given voting data based on the predicted average voting. Furthermore, Kim et al. [20] proposed a framework of a personal comfort model, which uses the Internet of Things and machine learning to learn personal comfort requirements directly from the data collected in the daily environment, and then aggregates the results to predict personal comfort. As poor indoor air quality will affect students’ comfort and attention levels, and research on indoor air quality mainly focuses on temperature, humidity, CO_2_, and PM_2.5_. Sharma et al. [21] proposed an IndoAirSense indoor air quality assessment and prediction framework, where multilayer perceptron (MLP) and eXtream gradient boosting regression (XGBR) are used to estimate the air quality of other rooms without sensors in real time. Finally, the improved long-term and short-term memory (LSTM) machine learning model is used to predict the air quality of rooms. In addition, Malkawi et al. [22] and Heibati et al. [23] proposed integrated simulation models where the control variables could be exchanged between them to assess the performance of energy consumption and indoor air quality.

While machine learning methods have gained increasing attention in recent years, the conventional models and algorithms have not yet incorporated the effect of interzonal airflow and boundary conditions into the data-driven predictions. In addition, training data-driven models requires a large amount of validated data from CFD simulation, but the creation of analytical models for CFD is computationally demanding. It is also time consuming to set up the simulation models, including the geometric information of building components (e.g., structural element dimensions and the locations and dimensions of windows), the thermal properties of building materials, the environmental conditions outside (e.g., solar radiation and wind), and the room occupancy [24]. According to the reviewed studies, the above-mentioned factors are usually manually acquired, including field measurements of the boundary conditions, physical tests of material surface concentrations and ventilation conditions, and data collection of daily environments, which heavily increases the difficulty of a CFD simulation setup given the limited accessibility of required building information. Therefore, the existing manual-based approach of simulation setup must be improved to efficiently obtain necessary data from an integrated data source.

Building information modelling (BIM), which becomes increasingly popular in the architecture, engineering, and construction industries, can provide detailed physical and functional characteristics of buildings [25]. To efficiently obtain accurate simulations of natural ventilation inside buildings, some studies have tried to combine BIM with CFD to conduct simulations, such as assessing the outdoor wind environment in the design stage of a building project using a BIM-based CFD tool [26], evaluating the building sustainability by integrating the BIM platform with CFD [27,28,29,30,31], and assessing natural ventilation by utilizing CFD and BIM. In the process of architectural design, most application strategies require careful consideration of indoor air quality and thermal conditions, but data interaction needs to be resolved during the design process. Therefore, previous researches proposed to use the BIM model as the basic platform for data interaction and obtain indoor comfort conditions through CFD of natural ventilation. Here, BIM provides two added values. First, BIM has rich building geometric and semantic information that can be conveniently extracted to set up computational domains and boundary conditions in CFD, which substantially improve the efficiency of simulation. In addition, the spatial information from design BIM models can be used to quantify the zone adjacency and connectivity and incorporate the potential impact of interzonal airflow in thermal comfort analysis. Both strengths provide advantages to characterize the input features for constructing more robust data-driven models.

Therefore, compared with the above-mentioned simulation models, this study aims to explore a BIM-enabled data-driven framework to underpin the predictive analysis of thermal comfort for indoor environments. With the proposed new framework, thermal comfort prediction can be examined more efficiently to study different design options, operating scenarios, and changeover strategies between various ventilation modes, such as better spatial HVAC system designs, specific room-based real-time HVAC control, and other potential applications, to maximize indoor thermal comfort. In this study, the rich building geometric and semantic information from design BIM models are extracted to set up simulation computational environments in CFD. In the neural network models, an adjacency matrix in the field of graph theory is used to represent the spatial features (such as zone adjacency and connectivity) and incorporate the potential impact of interzonal airflow in thermal comfort analysis. BIM data and CFD simulation results are then utilized to create data-driven models for predictive analysis of optimum thermal comfort. In addition, the deep neural network for thermal comfort forecasting is characterized in the analysis. The proposed new framework is applied in a case study to analyze the effect of air ventilation on the thermal comfort of an apartment. The results indicate that the proposed BIM-based and data-driven framework strengthens the predictive analysis of thermal comfort in the built environment. The rest of this paper is organized as follows. Section 2 is the methodology, which introduces the proposed BIM-supported framework. Section 3 applies an example to illustrate the proposed framework with detailed discussions on the predictive capacity of the deep neural network. Section 4 concludes the paper and describes the future work.

## 2. Materials and Methods

**Figure 1:**
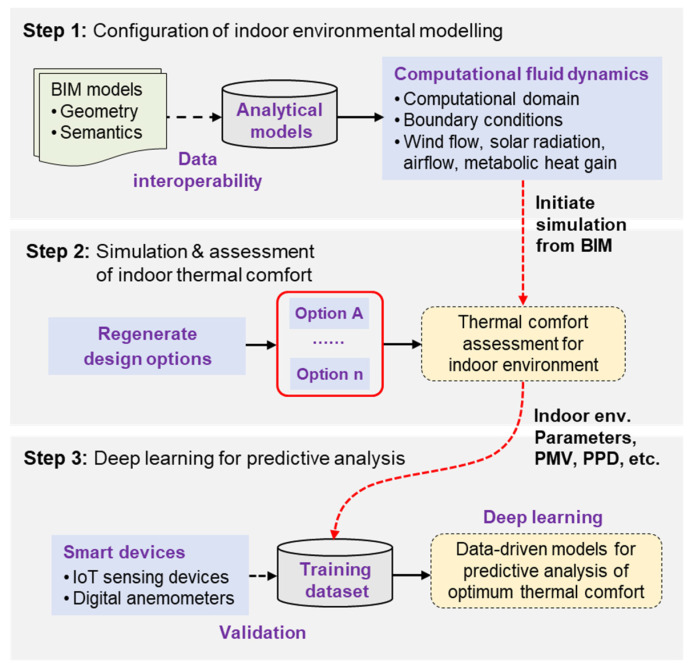
Proposed BIM-enabled data-driven framework for predictive analysis and optimization of thermal comfort.

Figure 1 shows the proposed framework that utilizes BIM-supported and data-driven methods for the numerical prediction of occupant thermal comfort. The framework consists of three parts: (1) configuration of indoor environmental modelling, (2) simulation and assessment of indoor thermal comfort, and (3) deep learning for predictive analysis. In the first part, indoor environmental modelling is configured by using BIM to provide the geometrical and topological attributes of building components (such as slabs, walls, and columns) as well as their material properties (e.g., solar transmittance, emissivity, and conductivity). The information is then used to generate the analytical model, including the simulation boundary condition and computational domain, for conducting CFD.

This is followed by simulation and assessment of indoor thermal comfort. It involves the determination of the operating status of mechanical air conditioning and the changeover between mechanical and natural ventilation. Different scenarios of design options can be generated for the indoor environment modelling and assessment of thermal comfort. For example, the CFD simulation of indoor airflow should emphasize the ventilation of natural airflow with windows being the inlet. The indoor air velocity and temperature from CFD simulation are utilized to evaluate Predicted Mean Vote (PMV) and Predicted Percentage Dissatisfied (PPD) for measuring the occupant comfort level.

Here, conduction, solar radiation, and internal heat sources contribute to increase the cooling load, potentially increasing the indoor temperature. In contrast, natural ventilation may increase or decrease the cooling load, depending on the difference between the indoor and outdoor temperature. If the outdoor temperature is lower than the indoor, natural ventilation may be sufficient to provide a cooling effect to maintain indoor temperature, and therefore mechanical ventilation can be turned off for energy savings. Otherwise, if the cooling load is more than the heat carried away by natural ventilation, mechanical ventilation is needed to maintain occupant thermal comfort, and CFD should focus on mechanical ventilation instead.

The final step of the proposed framework is to leverage the information from BIM–CFD to create data-driven models for the predictive analysis of optimum thermal comfort. Here, BIM technology not only provides the spatial features, but also the boundary conditions, indoor environmental parameters (indoor air velocity and temperature), PMV, and PPD through the CFD simulation process. These data are used in conjunction with relative humidity and personal inputs (i.e., activity level and clothing) to constitute the training dataset. In this analysis, the dataset was validated with smart devices such as IoT sensing devices or digital anemometers. The validated data were used to train machine learning data-driven models for predicting thermal comfort level and identifying the optimum design options and/or operating scenarios.

### 2.1. Configuration of Indoor Environmental Modelling

#### BIM-Enabled CFD Modelling of Indoor Environment

The BIM model provides the geometrical and topological details of building components and their material properties, which are extracted to create the simulation boundary conditions and computational domain for CFD. CFD simulation of an indoor environment was performed to evaluate the indoor airflow and temperature to support the occupant thermal comfort analysis at different periods. Equations (1) and (2) show the governing equations of the conservation of mass and momentum for modelling the motion of the fluid:

(1) $$\frac{\partial \rho }{\partial t}+\frac{\partial \left(\rho u_{i}\right)}{\partial x_{i}}=0$$

(2) $$\frac{\partial \rho u_{i}}{\partial t}+\frac{\partial \left(\rho u_{i}u_{j}\right)}{\partial x_{j}}=-\frac{\partial P}{\partial x_{i}}+\frac{\partial }{\partial x_{j}}\left[\mu \left(\frac{\partial u_{i}}{\partial x_{j}}+\frac{\partial u_{j}}{\partial x_{i}}\right)\right]$$

The simulation can be conducted for either mechanical or natural ventilation, based on the given scenarios or modelling of the heat transfer between indoor and outdoor environments. Boundary conditions for CFD simulation are obtained from sensor measurement, including the building layout, material properties, inlet airflow rate, air temperature, etc.

### 2.2. Simulation and Assessment of Indoor Thermal Comfort

#### 2.2.1. Heat Transfer of Natural Ventilation

As mentioned previously, different scenarios of design options can be generated for the indoor environment modelling and assessment of thermal comfort. This may involve the determination of the operating status of mechanical air conditioning and the changeover between mechanical and natural ventilation for CFD modelling. Equations (3)–(6) represent a simplified static method used in the building energy simulation program to determine the status of mechanical or natural ventilation. If the effect of natural ventilation can offset the heat gain from conduction, solar radiation, etc., the CFD simulation can emphasize the ventilation of natural airflow. Otherwise, mechanical ventilation is needed and thus CFD shall focus on the modelling of mechanical ventilation.

The hourly cooling load is calculated from the summation of heat gain through conduction, solar radiation, and internal heat sources as follows:

(3) $$Q\left(t\right)=\overset{K}{\underset{k=1}{\sum }}CQ_{k}^{\;}\left(t\right)+\overset{L}{\underset{l=1}{\sum }}SQ_{l}^{\;}\left(t\right)+\overset{M}{\underset{m=1}{\sum }}IQ_{m}^{\;}\left(t\right)\;\leq \;\overset{N}{\underset{n=1}{\sum }}NQ_{n}^{\;}\left(t\right)$$

(4) $$AC\left(t\right)=\{\begin{array}{l} 1,Q\left(t\right)\geq \overset{N}{\underset{n=1}{\sum }}NQ_{n}^{}\left(t\right)\\ 0,\mathrm{Otherwise} \end{array}$$

in which Q(t) represents the hourly cooling load (kWh) required to maintain the thermoset temperature at time *t*, $CQ_{k}^{\;}\left(t\right)$ refers to the heat conduction via an opaque or transparent surface *k* (kWh), $SQ_{l}^{\;}\left(t\right)$ stands for the solar radiation via a transparent surface *l* (kWh), $IQ_{m}^{\;}\left(t\right)$ represents the internal heat gain *m* (kWh), and $NQ_{n}^{\;}\left(t\right)$ is the heat transfer by natural ventilation via an opening *n* (kWh). AC(t) refers to the operating status of mechanical ventilation at time *t* (if the value is equal to one, mechanical ventilation should be utilized; otherwise, it should be turned off). If the cooling load from conduction, solar radiation, and the internal heat source is less than the heat taken away by natural ventilation, natural ventilation is sufficient to offset the heat gain from the ambient environment.

Natural ventilation is related to volumetric flow rate driven by wind and buoyancy as well as the difference between indoor and outdoor temperatures. Specifically, the heat flow due to the ventilation of air between the interior of a building and the exterior environment depends on the air exchange rate, as follows:

(5) $$NQ_{u}^{\;}\left(t\right)=C_{T}\rho_{air}V\left(t\right)\cdot \left(T_{out}-T_{in}\right)$$

(6) $$V\left(t\right)=A\cdot_{\;}{\left(\frac{2}{\rho_{air}}\left(P_{wind}+P_{buoyancy}\right)\right)}^{2}$$

where $NQ_{u}^{\;}\left(t\right)$ refers to the heat taken away by natural ventilation via an opening u at time t,$\;C_{T}$ is the specific heat coefficient (J/kg·°C),$\;\rho_{air}$ refers to the air density (kg/m^3^), V(t) stands for the volumetric flow rate (m^3^/s), $T_{out}$ is the outdoor temperature (°C), and $T_{in}$ is the indoor thermostat temperature (°C). *A* refers to the area of a window opening, and $P_{wind}\;\mathrm{and}\;P_{buoyancy}$ are the wind-driven and buoyancy-driven pressure, respectively. The availability and efficiency of natural ventilation due to different mixed-mode changeover strategies can be studied by varying the amount of V(t). The air velocity and temperature from CFD simulation (corresponding to different mechanical or natural ventilation scenarios) can be further utilized to assess the thermal comfort level.

#### 2.2.2. Assessment of Indoor Thermal Comfort Evaluation

Following the CFD simulation, a set of parameters can be obtained to evaluate thermal comfort. In this study, the Predicted Mean Vote (PMV) model [32] was used to measure thermal comfort, taking into consideration the three physical variables from CFD (i.e., air temperature, air velocity, and mean radiant temperature), one sensor measurement datum (i.e., relative humidity, RH), and two personal inputs (i.e., activity level and clothing), as follows:

(7) $$|PMV|=\left[0.303\cdot \exp{\left(-0.036\cdot M\right)}^{\;}+0.028\right]\cdot L$$

(8) $$\begin{array}{l} L=\left(M-W\right)-3.05\times 10^{-3}\cdot \left(5733-6.99\cdot \left(M-W\right)-Pa\right)-0.42\\ \;\cdot \left(M-2-58.15\right)-1.7\times 10^{-5}\cdot \left(5867-Pa\right)-0.0014\;M\\ \;\cdot \left(34-t_{a}\right)-3.96\times 10^{-8}f_{cl}\left[{\left(t_{cl}+273\right)}^{4}-{\left(\bar{t_{r}}+273\right)}^{4}\right]\\ \;-f_{cl}h_{c}\left(t_{cl}-t_{a}\right) \end{array}$$

in which *M* (W/m^2^) represents the metabolic rate, *L* (W/m^2^) refers to the heat transfer around a single building occupant, and *W* (W/m^2^) stands for effective mechanical power excreted by the occupant. *P_a_* (Pascals) is the water vapor partial pressure, which is related to the humidity level, *t_a_* (°C) is the air temperature, f_cl_ is the clothing surface area factor, which is dimensionless, *t_cl_* (°C) is the clothing surface temperature, $\bar{t_{r}}$ (°C) is the mean radiant temperature, and $h_{c}$ (W/(m^2^∙K)) is the convective heat transfer coefficient. The PMV model adopts a heat balance approach to evaluate the heat generation and loss to the environment. Specifically, (M−W) refers to the heat generated due to metabolism, while the remaining terms in Equation (8) stand for the heat transfer through skin, latent respiration, convection, radiation, etc.

In addition to PMV, Predicted Percentage Dissatisfied (PPD) is commonly used to quantitatively measure of the percentage of thermal discomfort for a group of building occupants at the indoor environment. PPD follows the below formula to calculate the thermal comfort:

(9) $$\mathrm{PPD}=100-95\cdot \exp\left[-\left(0.03353\cdot {\mathrm{PMV}}^{4}+0.2179\cdot {\mathrm{PMV}}^{2}\right)\right]$$

Table 1 shows the occupant comfort level based on different PMV grades and their approximate PPD that are used in the analysis.

#### 2.2.3. Regenerate Design Options

In this study, different design options were generated to assess thermal comfort using mechanical and natural ventilation. The configurations of the design BIM model were modified iteratively for CFD simulations. The flow field and temperature distribution obtained from CFD simulation were utilized to compute the PMV and PPD.

**Figure 2:**
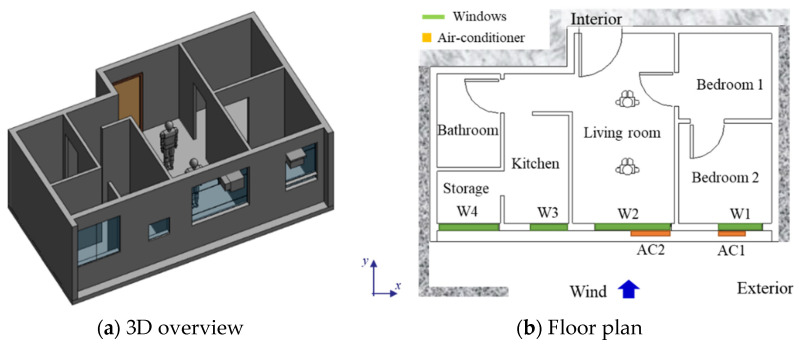
(**a**) The 3D overview and (**b**) floor plan for the parametric model used in analysis.

A typical apartment for residential buildings (see Figure 2) was created in BIM authoring software for applying the proposed framework. Figure 2 shows the 3D overview and floor plan for the parametric model used in analysis. The residential apartment in Hong Kong is designed for a couple with child(ren), consisting of two bedrooms (varying sizes), one living room, one kitchen, one bathroom, and one storage room. There are two air conditioning units: one installed in one of the bedrooms (AC1) and another in the living room (AC2). There are four windows facing the same direction with different sizes (W1, W2, W3, and W4). Table 2 summarizes the configuration of windows in the design BIM model. The computational boundaries for the simulation (such as the properties of concrete and glass) are input in design BIM models accordingly. For example, the thicknesses of concrete and glass are set as 200 mm and 6 mm, respectively. The density of concrete is 2400 kg/m^3^, while for glass the density is set as 2500 kg/m^3^. Another important parameter is thermal conductivity, which is set as 2.16 W/(m∙K) for concrete and 1.09 W/(m∙K) for glass.

The CFD simulations were conducted using Autodesk CFD [33], which accepts the geometry and semantics of the BIM model in SAT format exported from Autodesk Revit [34]. In this study, four boundary conditions in BIM models were identified to reflect different measures aiming to optimize the indoor thermal environment and human comfort. As Table 3 shows, mechanical and natural ventilation at different seasons were studied to explore the changeover strategy of the ventilation mode. CFD simulations for a typical night period of 7 PM in April and July (with the lowest and highest outdoor temperature, respectively) were conducted to analyze the effect of natural ventilation. This is because April has the lowest temperature due to the least solar heat gain during the daytime, whereas July normally has the highest ambient temperature.

**Figure 3:**
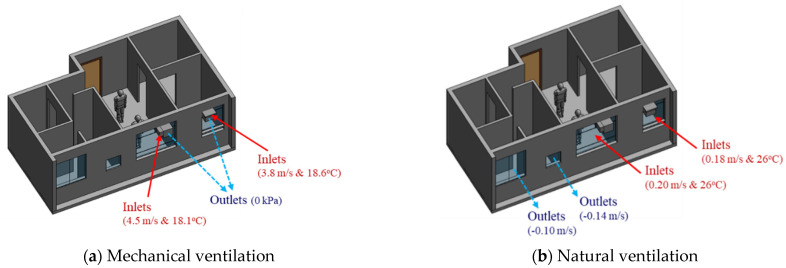
Parametric models from BIM and their boundary conditions for simulating (**a**) mechanical ventilation and (**b**) natural ventilation.

- The Baseline 1 scenario considers mechanical ventilation during a July night. The exterior walls are C40 concrete with 200 mm thickness (thermal conductivity = 2.16 W/(m∙K)). For the CFD simulation concerning mechanical ventilation, the ducts of air conditioning devices are defined as the inlets, whereas the vents are defined as the outlets (see Figure 3a). Digital sensor measurements were performed on inlets and outlets for the mechanical ventilation scenario. The measured airflow velocities of 4.5 m/s and 3.8 m/s with supplied air temperatures of 18.1 °C and 18.6 °C, respectively, are used in the mechanical ventilation simulation.
- The Baseline 2 scenario considers natural ventilation during a July night. The boundary conditions are similar to the Baseline 1 scenario, except that the supplied airflow and temperature at windows (that is, the inlets of natural ventilation) are measured from anemometers or sensing devices at 0.03–0.5 m/s respectively (see Figure 3b). For natural ventilation, the windows normal to the wind direction are defined as inlets with an airflow rate.
- Alternative 1 and 2 scenarios with different boundary conditions are conducted. Alternatives 1 assumes that the exterior wall is covered by an insulation layer of 50 mm, resulting in a decreased thermal conductivity of 0.16 W/(m∙K) for the composite wall system. The supplied airflow and temperature at windows are measured from anemometers or sensing devices at 0.03–0.5 m/s. The Alternative 2 scenario assumes natural ventilation during an April night, but the exterior wall has not been covered by insulation materials. The outdoor temperature decreases substantially to 19.5 °C.

Since the conventional heat loads from exterior walls and windows are difficult to measure, energy on the entire floor was assumed to be conserved to deduce reasonable values of heat flux on walls. Conservation of energy means that there is no energy loss in the system and hence the incoming heat has to equal the outgoing heat of the system. The incoming heat includes the inlet air temperature as well as the external and internal heat sources on the flat, whereas the outgoing heat consists of the outlet air temperature. The inlet and outlet temperature were obtained from digital sensor measurements. The external heat source is identified as heat flux on walls, while the internal heat loads are lamps and occupants. In the simulation, the heat load emitted from lamps was provided by light bulb specifications, while 100 W was assumed to be emitted by each occupant for performing sedentary work.

The parametric model from BIM is converted into the analytical model in CFD, wherein the solution domain is the air volume within the model. The exterior walls constitute the boundary of the computation domain. CFD uses the finite element method for discretization. The unstructured mesh with meshing size of 3,451,004 is used in this study to discretize the computational domain in CFD. The governing equations for fluid flow and heat transfer are the Navier–Stokes equations and the energy equation, respectively. The advection scheme used is Modified Petrov–Galerkin, a default streamline upwind advection scheme used by Autodesk CFD. To simulate turbulent flows, the SST k–ω turbulence model is used in this study. With the use of a blending function, this model takes advantage of both standard k–ε and standard k–ω turbulence models such that k–ε is used in freestreams while k–ω is used in near-wall regions. The temperature distribution and flow field obtained from CFD simulation are utilized to determine the PMV and PPD for training the deep learning models.

### 2.3. Deep Learning for Predictive Analysis

#### 2.3.1. Dataset Preparation

BIM–CFD simulation provides data that can be used to train data-driven models for the predictive analysis of occupant thermal comfort. This dataset, which covers 15,936 samples, is comprised of several key features: (1) spatial features, (2) boundary conditions, (3) indoor environmental parameters (air velocity, air temperature, and mean radiant temperature from CFD), (4) RH from digital sensor measurement, and (5) personal inputs based on different assumption (i.e., activity level and clothing). The performance of *PMV* and *PPD*, computed from Fanger’s model using CFD results, are labelled as the outputs. In this paper, c is defined to represent each data sample, whereas e and k stand for the specific feature and labelled output, respectively. Then, ${\left[\gamma_{c}^{\;}\right]}^{e}$ represents the value of a specific feature e for data sample c, and$\;{\left[\alpha_{c}^{\;}\right]}^{k}$ is the value of a labelled output k for data sample c.

In this study, the training dataset was split into two subsets in a K-fold validation—parts of the data were used for training, while the rest were used for validation in 10 iterative steps. An average value of prediction accuracy was used to evaluate the robustness of the model. In addition, a separate testing dataset with 664 data samples was used to test the accuracy of the models. The dataset information represents input features with different units that have large variances with various orders of magnitude, which may lead to poor prediction accuracy. As such, all the input features e and labelled outputs k are normalised within the range of 0 to 1 by using the min–max normalization, as follows:

(10) $$\tilde{\gamma_{c}^{\;}}=\frac{\gamma_{c}^{\;}-min\left(\gamma_{c}^{\;}\right)}{max\left(\gamma_{c}^{\;}\right)-min\left(\gamma_{c}^{\;}\right)}$$

(11) $$\tilde{\alpha_{c}^{\;}}=\frac{\alpha_{c}^{\;}-\min\left(\alpha_{c}^{\;}\right)}{\max\left(\alpha_{c}^{\;}\right)-\min\left(\alpha_{c}^{\;}\right)}$$

where $\gamma_{c}^{\;}$ and $\alpha_{c}^{\;}$ refer to the value of input features and labelled outputs, and $\tilde{\gamma_{c}^{\;}}$ and $\tilde{\alpha_{c}^{\;}}$ are the normalised values. The data preprocessing can avoid the dominance of a particular feature or output, and facilitate the convergence of gradient descent.

#### 2.3.2. Data-Driven Model

**Figure 4:**
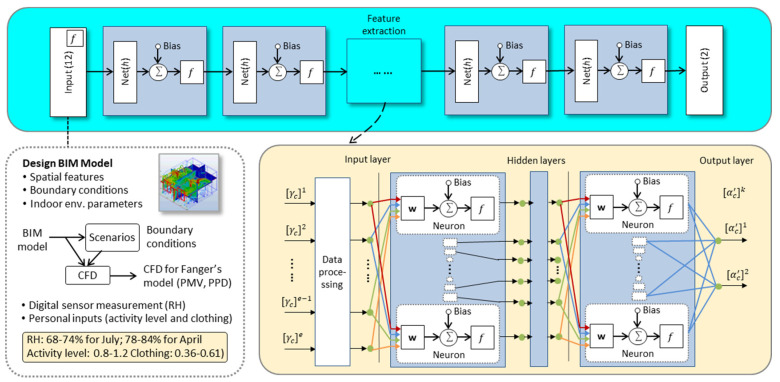
Data-driven model for predictive analysis of thermal comfort.

A multilayer neural network with hidden units can approximate any high-order, nonlinear function. With deeper, nonlinear hidden layers, deep neural network models can estimate complex nonlinear relationships based on data in comparison to conventional data-driven models. As shown in Figure 4, a set of input features e was defined first for each data sample c. This included (1) spatial features, such as size of the zone (Zone_Size), zone adjacency with interzonal flow impact (Interzonal_Flow), and coordinates (X, Y, Z), where the data sample was taken from BIM models. Zone adjacency can be represented by a graph structure, in which vertexes and edges represent zones and their connectivity, respectively. The graph structure can be transformed into a matrix form, where A(G) represents the adjacency matrix of the graph.

(12) $$A(G)=\left[\begin{array}{ccc} e_{1,1} & \cdots & e_{1,s}\\ \vdots & \ddots & \vdots \\ e_{r,1} & \cdots & e_{r,s} \end{array}\right]$$

Because the simple adjacency matrix only represents the space organization, weighting factors ($e_{r,s}$) are defined in A(G) to reflect the potential influence of interzonal airflow for thermal comfort. In this analysis, the weighting factors ($e_{r,s}$) are multiplied by connectivity ($H_{r,s}$), which represents the connectedness of a zone with its neighbouring zones.

(13) $$IA_{r}=\overset{S}{\underset{s=1}{\sum }}e_{r,s}\cdot H_{r,s}\;\;\;\;\;\;\;\;\;\;\;\;\;\;\;\;\;\forall r=1,2,\ldots ,R$$

Therefore, the interzonal airflow impact in this analysis is characterized by the potential influence of interzonal airflow for a zone and the zone connectivity. Another set of input features includes (2) boundary conditions, such as the number (No_Inlet), area (Inlet_Area), airflow (Inlet_Flow), and air temperature (Inlet_Tem) of the inlet, which can be taken from the BIM models. Following this, the input features also include (3) indoor environmental parameters such as air velocity, air temperature, and mean radiant temperature computed from CFD simulation. Other important features include (4) RH from digital sensor measurement, and (5) personal inputs, which include activity level (Activity_Level) and clothing (Clothing).

The output layer of the neural network has two neurons to provide *PMV* and *PPD*. The prediction results are compared with ground truth from Fanger’s model using CFD simulation results. The loss function can then be formulated, as follows:

(14) $$\mathrm{Loss}\;\mathrm{function}:\;L\left(w\right)=\sqrt{\overset{N}{\underset{c=1}{\sum }}\frac{{\left(\alpha_{c}^{\prime }-\alpha_{c}\right)}^{2}}{N}}+\lambda \cdot {\left|\left|w\right|\right|}^{2}$$

in which $\alpha_{c}^{\prime }$ is the prediction result from the network, $\alpha_{c}$ is the ground truth, N is the size of the training dataset (c refers to each data sample), w refer to the network weights, and λ stand for the weight decay, which controls the relative importance of the two parts in the loss function. In this study, the network weights, weight decay, and other hyper parameters were optimized iteratively during the training process in order to minimize the loss function. A higher value of λ indicates more importance of the regularization term and that the network performance is less sensitive to the predictions on the training dataset with better generalization performance on unseen data.

The number of layers and the number of neurons in each layer, etc., were reconfigured iteratively until the best predictions were found. These hidden units were updated during the learning process through backpropagation of every iteration. Fine-tuned by a series of experiments, a fully connected neural network provided reliable predictions and was therefore chosen in the subsequent analysis.

## 3. Results and Discussion

### 3.1. Comparison of BIM–CFD Simulation (Baseline 1) and Sensor Measurement

**Figure 5:**
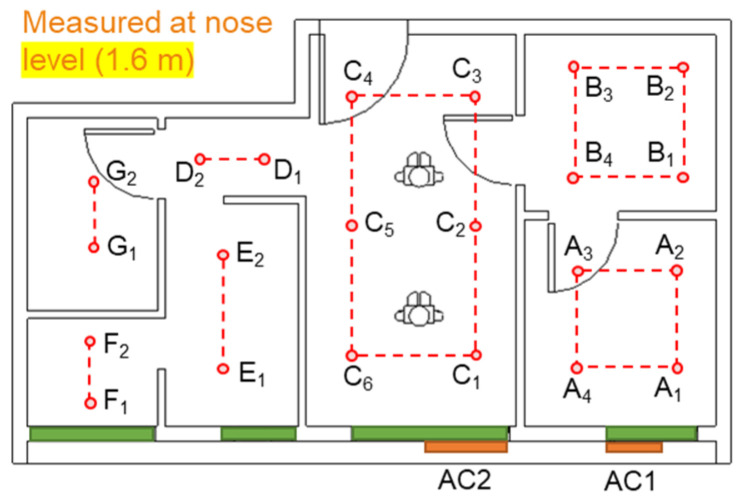
Data collection points for field measurement.

**Figure 6:**
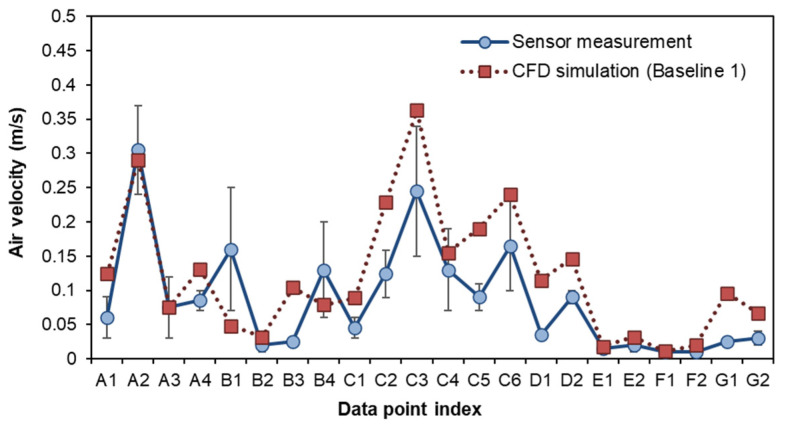
Comparison of airflow velocity from simulation and field measurement.

**Figure 7:**
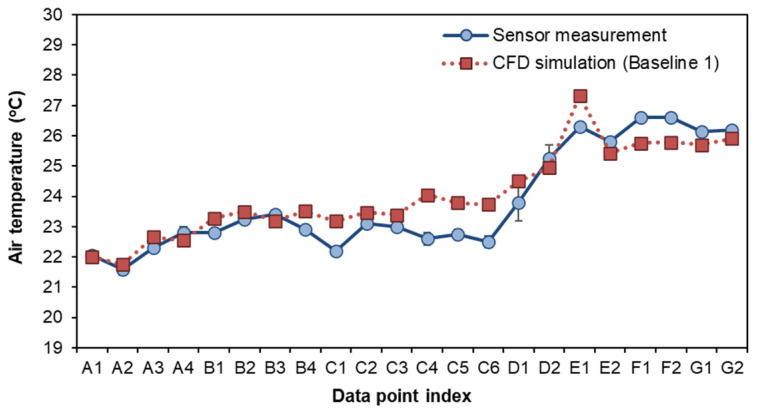
Comparison of indoor temperature from simulation and field measurement.

The simulation and measurement data were taken at nose level (around 1.6 m from the floor, 60% of storey height) for comparison. The locations of the data points were taken 0.4–0.6 m from the surfaces of the interior walls, as shown in Figure 5. The airflow rate and air temperature at each data collection point were measured 7–10 times, and the error variation of the measurement (e.g., average, minimum, and maximum) were recorded. As shown in Figure 6 and Figure 7, the indoor air velocity and temperature between simulated results (Baseline 1) and sensor measurements were compared. The error budgets are also marked in the Figures. According to the measurement, indoor airflow can be characterized by its low velocity (<0.5 m/s). The simulated air velocity results are close to the values measured in bedrooms, kitchen, and storage room.

As compared to the sensor measurement, the simulation slightly overestimates air velocity in the living room. The differences between the measured and simulated values are generally less than 0.1 m/s in air velocity for 18 out of 22 data points. As for air temperature, there is less than a 5% difference between measured and simulated values for 21 out of 22 data points. The indoor airflow is characterized by its very low velocity (<0.5 m/s) but a large fluctuation. The simulated results are higher in the living room and kitchen, but lower in the toilet. In particular, a greater error variation can be observed at the direct downflow and the circulated flow of air conditioning devices (such as A2, B1, B4, C3, C4, and C6). As compared to airflow rate, temperature distribution remains more stable. The field measurement is used to verify the simulated results. Since more than 80% of the simulated values have a small discrepancy with the measured data, the CFD results used in this study are considered acceptable for training the data-driven models.

### 3.2. Data Analysis

**Figure 8:**
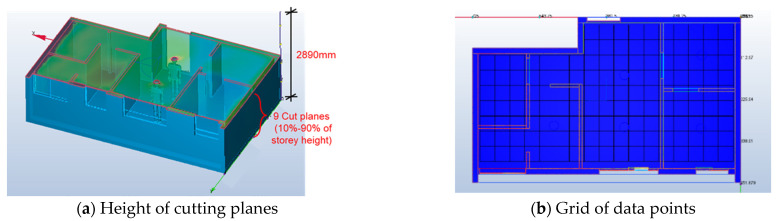
Cutting planes and data points.

**Figure 9:**
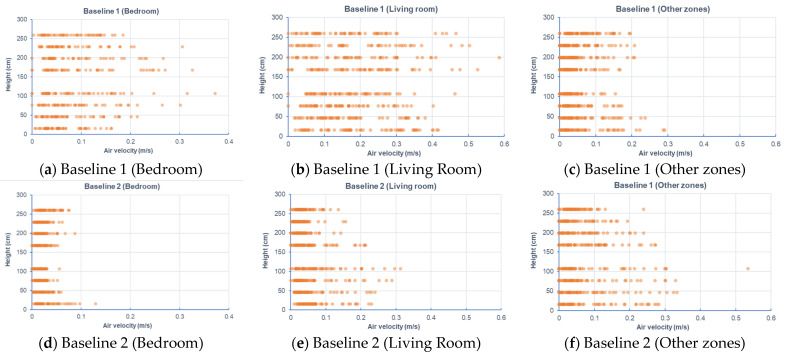

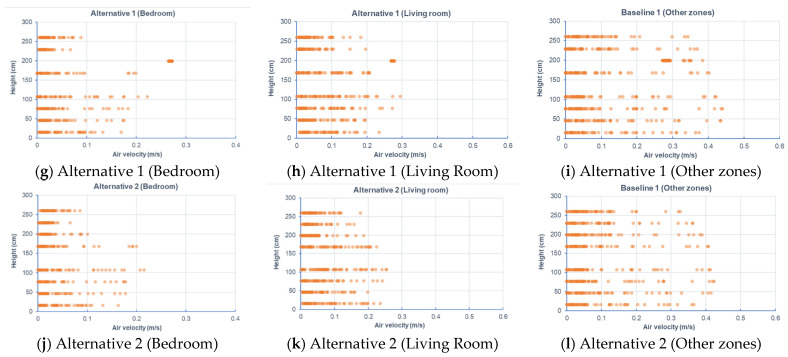
Air velocity for different rooms taken from different design options.

**Figure 10:**
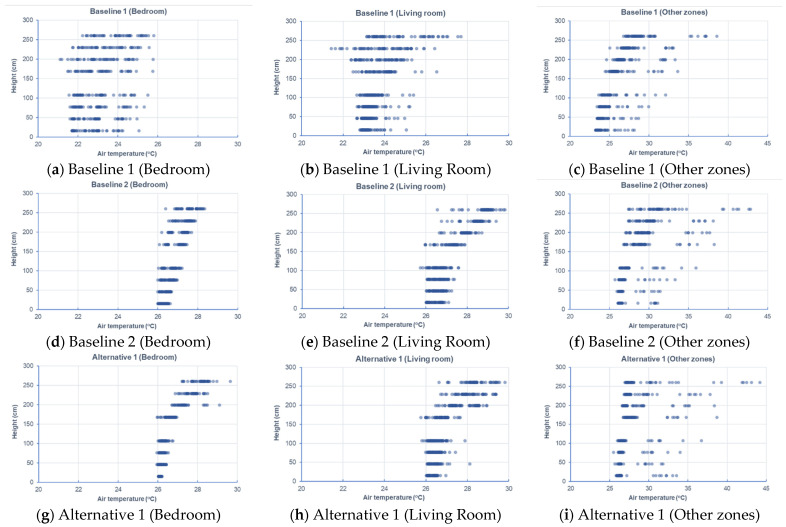

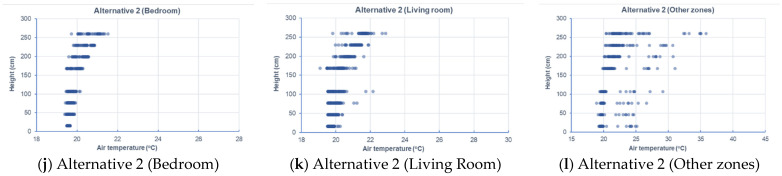
Air temperature for different rooms taken from different design options.

**Figure 11:**
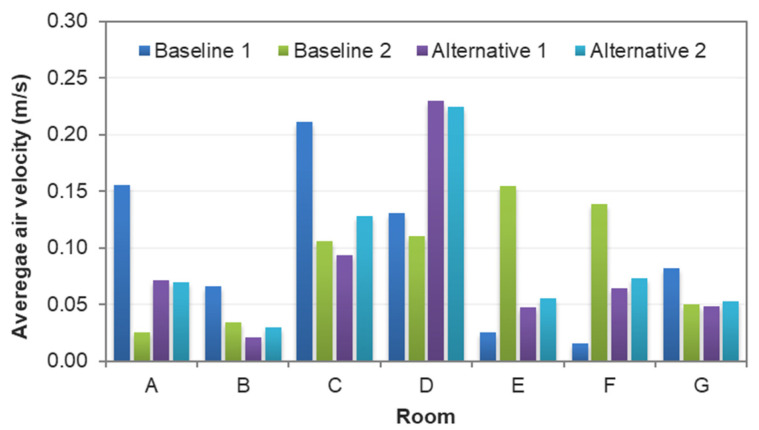
Comparison of average airflow velocity between different rooms.

**Figure 12:**
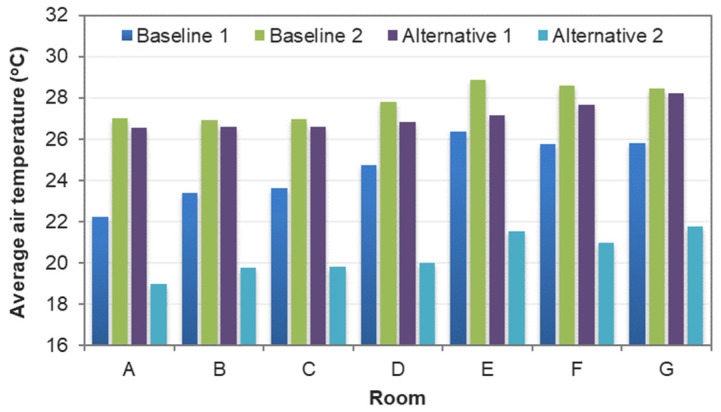
Comparison of average indoor temperature between different rooms.

**Figure 13:**
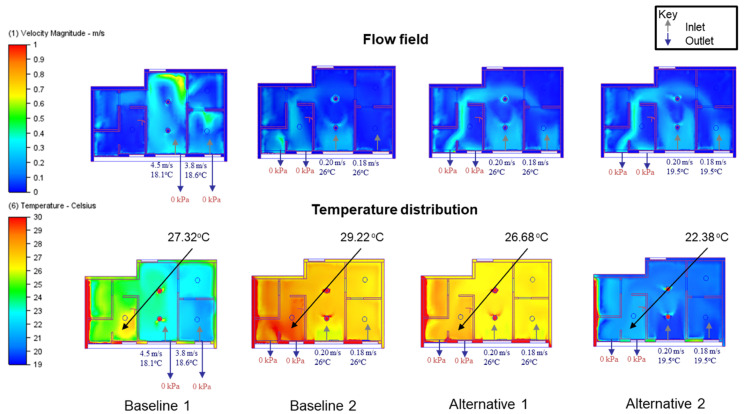
Comparison of CFD results between four design options (at nose level, around 1.6 m, 60% of storey height).

The CFD results were taken from nine cutting planes (varying from 10%, 20%, 30, 40%, 50%, 60%, 70%, 80%, and 90% of the storey height of 2890 mm), each of which had a grid of data points, as shown in Figure 8. A total of 5312 data points were defined in the design BIM model to automatically acquire the simulation results. With different variations of RH (68–74% for July; 78–84% for April), activity level (0.8, 1.0, 1.2), and clothing (0.36, 0.54, 0.61), a total of 15,936 samples were prepared to constitute the training dataset. Figure 9 and Figure 10 show the air velocity and temperature for different rooms taken from the four design options. Figure 11 and Figure 12 compare the average air velocity and temperature for different rooms (in accordance with the data point index in Figure 5). Figure 13 visualizes the flow field and temperature distribution.

For all scenarios, the air change rate for most of the rooms/zones was below 0.4 m/s. The local air draft was hence minimal. The airflow patterns were induced by air conditioners in the case with mechanical ventilation and windows in the cases with natural ventilation.

- The case with mechanical ventilation, which is Baseline 1, has higher air velocities in the bedrooms and living room (see Figure 9a–c). The apartment has a comfortable air change rate, as the air velocity for bedrooms, living room, and other zones are generally in the range of 0–0.2 m/s, 0–0.4 m/s, and 0–0.2 m/s, respectively. As Figure 10a–c shows, the temperature in bedrooms and living rooms is around 20–24 °C, and occupants may feel comfortable or slightly cool. In contrast, the air temperature in other zones (such as corridor, kitchen, etc.) is higher than 25 °C and even approaches 35 °C, which can lead to human discomfort.
- In the case of Baseline 2 when natural ventilation is utilized, the apartment has a comfortable air change rate as the air velocity for bedrooms, living room, and other zones are generally in the ranges of 0–0.1 m/s, 0–0.2 m/s, and 0–0.3 m/s, respectively (see Figure 9d–f). As compared to Baseline 1, lower air velocities are observed for bedrooms and living rooms, whereas higher air velocities are recorded for other zones like the kitchen and storage rooms, where windows are installed. This is more evident when comparing the data in Figure 11, Figure 12 and Figure 13. As can be seen in Figure 10d–f, the air temperature of bedrooms and the living room ranges mainly from 26 to 28 °C and 26 to 30 °C, while the temperature for the toilet, kitchen, and storage room is above 26 °C and reaches even 38 °C. The indoor environment of bedrooms and the living room is generally comfortable, while occupants staying long-term in other zones may feel warm and even hot.
- In the case of Alternative 1, the apartment shows a similar flow field and temperature distribution as Baseline 2 with a generally comfortable indoor environment. The temperature is well above 26 °C. As can be seen in Figure 12 (refer to E), the temperature rises to 29.22 °C in the kitchen for Baseline 2 (the case without insulation), which is too hot for the occupant to cook there. With Alternative 1 with insulation installed in the external walls, the temperature can decrease to below 27 °C. As compared to Baseline 1 when mechanical ventilation is utilized, the zones with the air conditioners installed have higher air velocity and lower air temperature than that in Alternative 1 (the adjacent zones without air conditioners installed).
- Alternative 2 assumes that natural ventilation is used in April when the outdoor air temperature is much lower than Baseline 2 and Alternative 1. While a similar pattern of air velocities is observed (Figure 9j–l), the temperature distribution varies significantly for different zones. As for the natural ventilation case at night in April, the overall temperature is much cooler and below 22 °C. As Figure 10j–l shows, air temperature ranges mainly from 18 to 22 °C for bedrooms and the living room, and 19 to 30 °C for other zones including the corridor, kitchen, and storage room. The occupant may feel mostly cool in bedrooms and the living room due to the low temperature, but comfortable at other zones depending on their activity level and clothing.

The results suggest that adopting natural ventilation is sometimes favorable because the indoor environment can be maintained as thermally comfortable as with air conditioners. Of note is that natural ventilation reduces the energy consumption due to the use of a mechanical system. If the occupants spend most of their time in bedrooms and the living room, switching off mechanical ventilation at night in July should be considered as a good practice, as this reduces energy consumption. Still, natural ventilation cannot always provide a comfortable indoor environment due to the weather profile in different seasons.

### 3.3. Performance of Data-Driven Models

**Figure 14:**
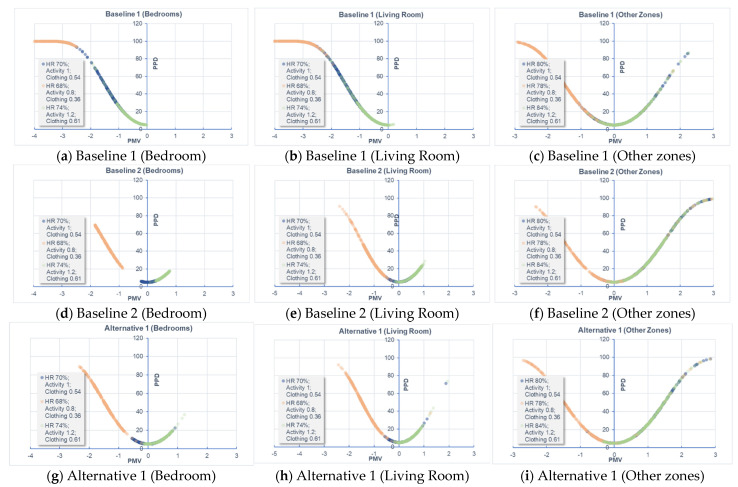

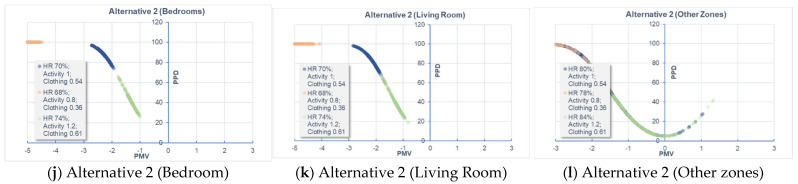
PMV versus PPD for different design options.

The comfort index, PMV and PPD, calculated from the CFD simulation is also important as the study aims to assess and identify the optimum thermal comfort. As discussed previously, a total of 15,936 samples were prepared to constitute the training dataset, with different variations of RH (68–74% for July; 78–84% for April), activity level (0.8, 1.0, 1.2) and clothing (0.36, 0.54, 0.61). Figure 14 shows the PMV versus PPD for different design options. When RH reaches over 70% and the indoor temperature increases over the neutral temperature range, the indoor environment tends to prevent sweat evaporation and therefore more easily leads to occupant discomfort.

In Baseline 1, the occupants who stay in bedrooms and the living room would feel comfortable if their activity level is 1.2 and clothing is 0.61 (HR = 74%). If the activity level and clothing decrease to 0.8 and 0.36, respectively, the occupants feel cool and even cold for a long-term stay. In the cases of Baseline 2 and Alternative 1, the air temperature in bedrooms and the living room is well above 26 °C and |PMV| is <0.3. Most of the occupants would feel comfortable to stay in the bedrooms and living room except for HR 74%, activity level 0.8, and clothing 0.36, and occupants who recline may feel slightly cool. As mentioned earlier on, the air temperature for Alternative 2 ranges mainly from 18 to 22 °C. Regardless of the activity level and clothing, the occupant may feel slightly cool and cold (especially in bedrooms and the living room) due to the low temperature. Although the thermal comfort index varies significantly for other zones such as kitchen, storage rooms, etc., occupants usually spend limited time in these areas, as the main living areas are bedrooms and living room.

**Figure 15:**
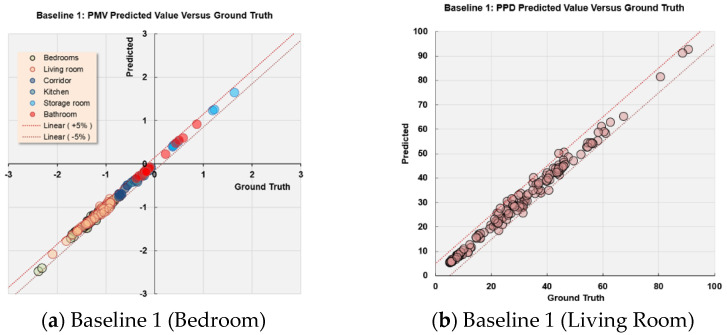

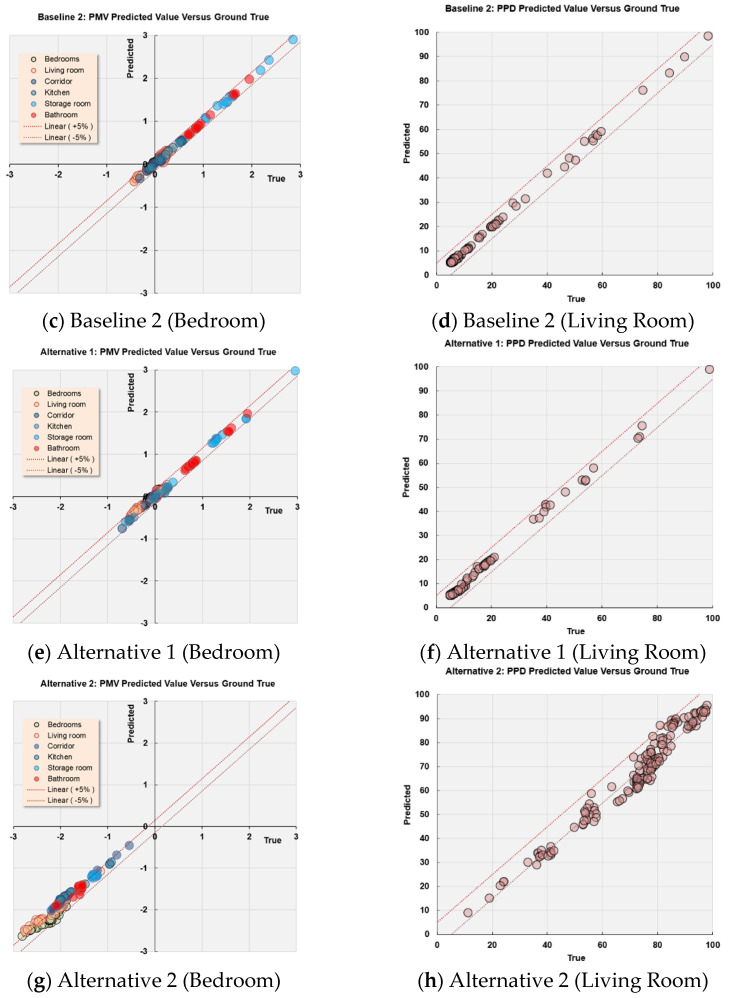
Predicted value versus ground truth for different design options.

The training dataset aforementioned were used to train the data-driven models using the proposed deep neural network in Section 2.3.2. The models exhibited good accuracy in predicting thermal comfort index. To quantify the prediction accuracy, new indicators are proposed to evaluate the improvement of the proposed algorithm over other alternatives:

(15) $$AE=\overset{N}{\underset{c=1}{\sum }}\left|\alpha_{c}^{\prime }-\alpha_{c}\right|\cdot N^{-1}\;\;\;\;\;\;\;\;\forall \;k=1,2$$

(16) $$APE=\overset{N}{\underset{c=1}{\sum }}\left|\frac{\alpha_{c}^{\prime }}{\alpha_{c}}-1\right|\cdot N^{-1}\;\;\;\;\;\;\;\;\forall \;k=1,2$$

in which AE and APE are the average error and percentage error. $\alpha_{c}^{\prime }$ is the prediction result from the network, $\alpha_{c}$ is the ground truth, N is the size of the training dataset (c refers to each data sample), and k (=1,2) refers to PMV and PPD, respectively. One thing of note is that neither of the models produces too closely of an analysis to the training dataset and therefore would be able to fit new inputs to predict reliably the output PMV and PPD. The trained data-driven models were tested on an extra 664 data samples in order to evaluate the model accuracy and flexibility in predicting thermal comfort index for a new set of input data. Figure 15 compares the prediction results versus the ground truth (obtained from Fanger’s model) for PMV and PPD, respectively. Here, the prediction results are reliable since the majority of the prediction errors do not exceed 5% errors.

Specifically, the average errors of PMV for Baselines 1 and 2 and Alternatives 1 and 2 are 0.03, 0.03, 0.04, and 0.13, respectively. For PPD, the average errors are 1.33%, 0.35%, 0.41%, and 5.52% for the four design options, respectively. Taking into consideration the normal distribution of PMV [−3, 3] and PPD [0, 100], the magnitude of prediction errors is acceptable. Alternative 2 has relatively greater errors because it assumes the use of natural ventilation in April when the outdoor air temperature (19.5 °C) in CFD simulation is much lower than other scenarios. At this season, the activity level and clothing for indoor occupants have large impacts on thermal comfort. This is more evident in Figure 14 where the PMV for Alternative 2 is more widely distributed than other scenarios. The large discrepancy of PMV and PPD for data samples may lead to poor predictive capacity of the data-driven models. Nevertheless, it can be clearly seen that the data-driven models exhibit good overall performance and accuracy between the predicted PMV/PPD against the ground truth. The data-driven models can be used for future predictive analysis of indoor environments toward identifying the optimum thermal comfort.

## 4. Conclusions

This paper presents a BIM-enabled and data-driven framework to underpin the assessment and predictive analysis of thermal comfort for an indoor environment. BIM technology provides 3D digital building models (with information on the building geometry, materials, and ambient built environment) that can be conveniently extracted for CFD simulation to prepare the training dataset. With BIM–CFD simulation results, a more reliable machine learning data-driven model can be developed for the predictive analysis of indoor thermal comfort. In the neural network, an adjacency matrix in the field of graph theory is used to represent the spatial features (such as zone adjacency and connectivity) and incorporate the potential impact of interzonal airflow in thermal comfort analysis. The results indicate that the average errors vary from 0.03 to 0.13 for PMV and from 0.35% to 5.52% for PPD, respectively. Taking into consideration the normal distribution of PMV [−3, 3] and PPD [0, 100], the magnitude of prediction errors is acceptable. With the proposed new framework, thermal comfort prediction can be examined more efficiently to study different design options, operating scenarios, and changeover strategies between various ventilation modes. For example, this prediction model could be used for exploring better spatial and HVAC system designs to maximize thermal comfort or for supporting real-time HVAC system controls for specific rooms of a building. In addition, facing the increasing development of a digital twin, the proposed BIM-based prediction model can be integrated with IoT to conduct predictive maintenance for avoiding unexpected thermal comfort interruption.

In the data analysis, indoor airflow is characterized by lower velocities but high fluctuations, varying significantly for different rooms/zones of a residential apartment. For living rooms and bedrooms where air conditioning devices directly supply the cooled air, the temperature can be maintained at a comfortable level. In contrast, the indoor temperature and airflow rate for kitchen and bathroom are higher than the suggested value from green building standards. Trade-off analysis regarding human comfort can help explore appropriate improvement measures, such as the increase of set point temperature, change of building envelope design, and enhancement of natural ventilation. The results also indicate that the data-driven models provide robust estimation of thermal comfort index, i.e., PMV and PPD. The proposed framework can be extended in the future by leveraging the cutting-edge graph convolutional neural network that can more efficiently characterize the space organization and indoor air features, then propagate the information in the neural network for predicting thermal comfort.

## Figures and Tables

**Table 1:** Occupant comfort level based on PMV and PPD.

Occupants’ Feeling	Cold	Cool	Slightly Cool	Neutral	Slightly Warm	Warm	Hot
PMV	−3	−2	−1	0	1	2	3
PPD	>93.5	76.9	26.3	5	26.3	76.9	>93.5

**Table 2:** Configurations of windows in design BIM model.

ID	W1 (Casement)	W2 (Casement)	W3 (Casement)	W4 (Sliding)
Size	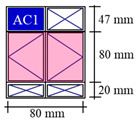	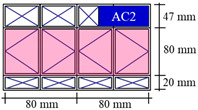	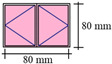	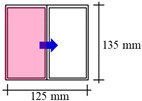

**Table 3:** Summary of boundary conditions for CFD simulation.

Scenarios	Ventilation Type	Time		Building Geometry	Thermal Conductivity of Exterior Wall (W/(m∙K))	Environmental Conditions from Sensor Measurement	
						Outdoor Temperature	Supplied Airflow and Temperature
Baseline 1	Mechanical	July	Night	Parametric models in BIM (refer to Figure 3)	2.16 (200 mm concrete)	26 °C	3.8 m/s and 18.6 °C (AC1) 4.5 m/s and 18.1 °C (AC2)
Bassline 2	Natural	July	Night		2.16 (200 mm concrete)	26 °C	0.03–0.5 m/s for different windows
Alternative 1	Natural	July	Night		0.16 (200 mm concrete and 50 mm insulation)	26 °C	0.03–0.5 m/s for different windows
Alternative 2	Natural	April	Night		2.16 (200 mm concrete)	19.5 °C	0.03–0.5 m/s for different windows

## Data Availability

The data presented in this study can be obtained on request from the correspondence. The data are not publicly available due to confidentiality reasons for our future studies.
